# Prognostic Value of a Clinical Biochemistry-Based Nomogram for Coronavirus Disease 2019

**DOI:** 10.3389/fmed.2020.597791

**Published:** 2021-01-18

**Authors:** Jing Yu, Lei Nie, Dongde Wu, Jian Chen, Zhifeng Yang, Ling Zhang, Dongqing Li, Xia Zhou

**Affiliations:** ^1^Department of Blood Transfusion, Tongji Medical College, Wuhan No.1 Hospital/Wuhan Hospital of Traditional Chinese and Western Medicine, Huazhong University of Science and Technology, Wuhan, China; ^2^Department of Hepatobiliary Pancreatic Surgery, Tongji Medical College, Hubei Cancer Hospital, Huazhong University of Science and Technology, Wuhan, China; ^3^Department of Head and Neck Surgery, Tongji Medical College, Hubei Cancer Hospital, Huazhong University of Science and Technology, Wuhan, China; ^4^Department of Thoracic Surgery, Jinyintan Hospital, Wuhan, China; ^5^Key Laboratory of Occupational Hazard Identification and Control in Hubei Province, School of Public Health, Wuhan University of Science and Technology, Wuhan, China; ^6^Department of Microbiology Medicine, Wuhan University of Basic Medical of Science, Wuhan, China; ^7^Department of Respiratory and Critical Care Medicine, Jinyintan Hospital, Wuhan, China

**Keywords:** Coronavirus disease 2019, inflammatory markers, carcinoembryonic antigen, hazard ratio, prognosis

## Abstract

**Background:** This study aimed to explore the predictive value of a clinical biochemistry-based nomogram in COVID-19.

**Methods:** The plasma or serum concentrations/levels of carcinoembryonic antigen (CEA) and other biomarkers, e.g., C-reactive protein (CRP), white blood cell (WBC), interleukin-6 (IL-6), ferritin (Fer), procalcitonin (PCT), lymphocyte percentage (L%), D-dimer (D2), and neutrophils percentage (Neu%), were assessed in 314 hospitalized patients with confirmed COVID-19. The area under the curve was used to estimate the diagnostic and prognostic value for COVID-19. Cox and logistic regression analyses were used to estimate the independent prognostic risk factors for the survival of patients with COVID-19.

**Results:** Receiver operating characteristic (ROC) curves were used to determine the area under the curve (AUC) values for CEA, IL-6, CRP, PCT, Fer, D-dimer levels and L%, Neu%, and WBC to assess disease classification. The critical values for these markers to predict severe disease type were then determined. The hazard ratio of prognosis for risk of COVID-19 identified CEA, WBC, CRP, PCT, Fer, D-dimer, Neu%, and L% as independent prognostic factors. For the nomogram of overall survival (OS), the C-index was 0.84, demonstrating a good discriminative performance.

**Conclusions:** An OS nomogram for the clinical diagnosis and treatment of COVID-19 was constructed using biomarkers. These data will be useful for the diagnosis, management, and therapy of COVID-19.

## HIGHLIGHTS

- We constructed an OS nomogram to diagnose and treat COVID-19, with a good C-index.
- CEA, WBC, CRP, PCT, Fer, D-dimer, Neu%, and L% were independent prognostic factors.
- The prognostic risk score identified high risk populations for OS.
- According to the hazard ratio for prognosis, we identified high risk factors for patient OS.

## Introduction

Coronavirus disease 2019 (COVID-19) has become a worldwide threat to human health. It is caused by infection with a virus known as severe acute respiratory syndrome coronavirus 2 (SARS-CoV-2) (1). Intensive efforts are being made to prevent and treat this disease. According to the seventh edition of the diagnostic and treatment guidelines for the novel coronavirus, the diagnosis of this disease has been linked to epidemiological history, typical chest computed tomography imaging features of COVID-19, and other etiological investigations (2). The levels of certain inflammatory biomarkers, such as C-reactive protein (CRP), lymphocyte (L) percentage, neutrophils percentage (Neu%), interleukin-6 (IL-6), procalcitonin (PCT), ferritin (Fer), D-dimer (D2), and the white blood cell (WBC) count, have been used to assess disease progression (3–5). Our previous study noted that the carcinoembryonic antigen (CEA) level is an independent prognostic marker for COVID-19 (6). In the present study, we aimed to explore the value of all the above markers to diagnose and predict the prognosis of COVID-19. In addition, we aimed to use these factors to construct and validate a nomogram to predict the overall survival (OS) of patients with COVID-19.

## Methods and Materials

### Patient Cohort

From January 24 to April 26, 2020, 314 patients infected with SARS-CoV-2 at Wuhan Jinyintan Hospital agreed to be included in this study. COVID-19 was confirmed in these patients based on characteristic manifestations on chest computed tomography (CT), etiological evidence, and epidemiological history (not including the presence of tumors). According to the seventh edition of the diagnosis and treatment plan for COVID-19 in China, the clinical conditions of patients with COVID-19 may be classified into four types: mildly affected, moderately affected, severely affected, and critically severely affected (2, 7). At the time of admission, the classification of the 314 patients was as follows: 83 cases had moderate symptoms with fever, CT manifestations, and respiratory distress; 155 cases showed severe symptoms; and 76 cases were critically severely affected, with acute respiratory distress syndrome. Throat swabs were collected from enrolled patients to detect SARS-CoV-2 RNA using real-time PCR with a Nucleic Acid Extraction Kit (8) (Zhijiang Orient Gene Biotechnology Company, Shanghai, Chins) and a 2019-nCoV ORFlab and N genes target detection kit (Zhijiang Orient Gene Biotechnology). The ethics committee of Jinyintan Hospital approved the study (Ethical approval number: KY-2020-69.01). The study was carried out in accordance with the current revision of the Declaration of Helsinki.

### Detection of CEA and Inflammatory Biomarkers

The serum levels of CEA and Fer were detected using a chemiluminescence immunoassay (Abbott Laboratories, Chicago, IL, USA) and their associated reagents, while the levels of CRP were detected using a biochemical analyzer (Abbott Laboratories). Blood counts were performed using a Mindray BC-6900 blood hematology analyzer (Mindray medical international limited, Shenzhen, China) and its associated reagents. The levels of IL-6 were detected using a Roche automatic electrochemiluminescence immunoassay and its associated reagents (Roche diagnostic Company limited, Basel, Switzerland). The PCT levels were assessed using a mini-Vidas immunofluorescence analyzer (BioMerieus Company, Craponne, France), The D-dimer level was assessed using a Stago automatic coagulometer (Stago diagnostic Company limited, Paris, France).

### Clinical Classification

All patients were clinically classified as follows (1, 9–11): (1) Mild: patients' clinical symptoms were mild, with no signs of pneumonia on CT scans; (2) Moderate: the patient has fever, respiratory tract symptoms, and signs of pneumonia on CT scans; (3) Severe: the patient met any of these criteria: shortness of breath, return rate (RR) over 30 times per min; an at-rest oxygen saturation (SpO2) level lower than 93%; partial pressure of arterial oxygen (PaO_2_)/the fraction of inspired oxygen (Fi02) lower than 300 mmHg (1 mmHg = 0.133 kpa); chest CT scans showing significant disease progression within 1 to 2 days; and (4) Critically severe: the patient met any of these criteria: respiratory failure requiring mechanical ventilation; shock; and complications related to organ failure that required ICU stay.

### Statistical Analysis

Statistical analysis was performed using SPSS version 20.0 (IBM Corp., Armonk, NY, USA). To analyze the differences in the levels of CEA, CRP, and other biomarkers among patients with COVID-19, the chi-square test and Kruskal-Wallis *H*-test were used. Univariate analysis and multivariate Cox regression were used to identify independent prognostic factors. The R software package (Version 3.4.4) was used to analyze the constructed nomograms for OS probability. To evaluate the specificity and sensitivity of the indicator levels to predict the severity of pneumonia, receiver operating characteristic (ROC) curves were used. Spearman's rank correlation significance test was used to analyze the association between individual patient variables. Statistical significance was accepted at *p* < 0.05.

## Results

### Demographic Characteristics of the Patients

Table 1 details the clinical characteristics of the included patients. Of the 314 patients, 83 showed moderate symptoms, 155 had severe symptoms, and 76 displayed critically severe symptoms at the time of admission. Of the 314 patients, 133 were female, and 181 were male. The patients' ages ranged from 35 to 91 years old, with a mean age of 64.65 years old. Around 52.87% (166) of the patients were over 65 years old. In our study, no significant differences in IL-6, CRP, PCT, or WBC counts by sex or age were observed (*P* > 0.05). However, the levels of CEA, D2, L%, and Neu% were higher in patients over 65 years old (*P* < 0.05), while have no significant differences in sex (*P* > 0.05).

**Table 1:** The clinical characteristics of 314 patients with COVID-19.

**Group**	***N***	**IL-6 (pg/ml)**	**WBC (× 10^**9**^/L)**	**L%**	**N%**	**CRP (mg/L)**	**PCT (ng/ml)**	**D2 (μg/ml)**	**CEA (ng/ml)**	**Fer (ng/ml)**
**Sex**										
Male	181	19.78 ± 2.95	9.86 ± 0.48	10.99 ± 0.71	82.19 ± 1.17	78.56 ± 5.33	0.99 ± 0.25	12.84 ± 2.36	13.63 ± 1.05	1192 ± 59.91
Female	133	12.49 ± 1.41	10.38 ± 0.57	13.19 ± 0.96	80.87 ± 1.22	68.49 ± 6.60	0.60 ± 0.25	11.35 ± 2.47	15.04 ± 1.16	742.7 ± 65.52
*P*-value		0.054	0.483	0.061	0.438	0.231	0.283	0.685	0.368	<0.001
**Age**										
≥65	166	18.64 ± 2.82	10.54 ± 0.51	10.55 ± 0.70	84.15 ± 0.95	79.06 ± 5.62	0.88 ± 0.25	15.71 ± 2.65	16.02 ± 1.20	1062 ± 62.59
<65	148	14.3 ± 2.07	9.52 ± 0.53	13.72 ± 0.95	78.31 ± 1.43	68.18 ± 6.19	0.74 ± 0.27	7.46 ± 1.75	12.12 ± 0.89	931.5 ± 70.43
*P*-value		0.251	0.171	0.007	0.001	0.194	0.698	0.020	0.012	0.166
**The admission classification**										
Moderate	83	16.81 ± 3.49	7.78 ± 0.49	17.5 ± 1.14	75.94 ± 1.35	48.34 ± 6.64	0.16 ± 0.06	2.99 ± 1.29	12.11 ± 1.21	704.8 ± 75.05
Severe	155	16.33 ± 2.55	10.2 ± 0.50	11.13 ± 0.75	82.18 ± 1.24	75.21 ± 5.55	0.83 ± 0.23	9.16 ± 1.71	14.78 ± 1.13	1088 ± 60.89
Critical severe	76	18.43 ± 4.47	12.91 ± 0.93	7.01 ± 0.89	88.27 ± 1.33	111.7 ± 9.84	1.97 ± 0.76	10.97 ± 2.65	16.01 ± 1.92	1342 ± 109.1
*P*-value		0.913*, 0.775^&^, 0.685^#^	0.002*, <0.001^&^, 0.008^#^	<0.001*, <0.001^&^, 0.003^#^	0.002*, <0.001^&^, <0.001^#^,	0.003*, <0.001^&^, 0.001^#^	0.037*, 0.002^&^, 0.056^#^	0.017*, 0.004^&^, 0.585^#^	0.127*, 0.073^&^, 0.569^#^	<0.001*, <0.001^&^, 0.041^#^

The ^*^ symbol represents the comparison of the moderately affected patients vs. the severely affected patients; the ^&^ symbol represents the comparison of the moderately affected patients vs. critically severely affected patients; the ^#^ symbol represents the comparison of the severely affected patients vs. the critically severely affected patient.

### Correlations Between CEA, IL-6, CRP, PCT, Fer, D-Dimer Levels, L%, Neu%, WBC, and Clinical Classification

**Figure 1:**
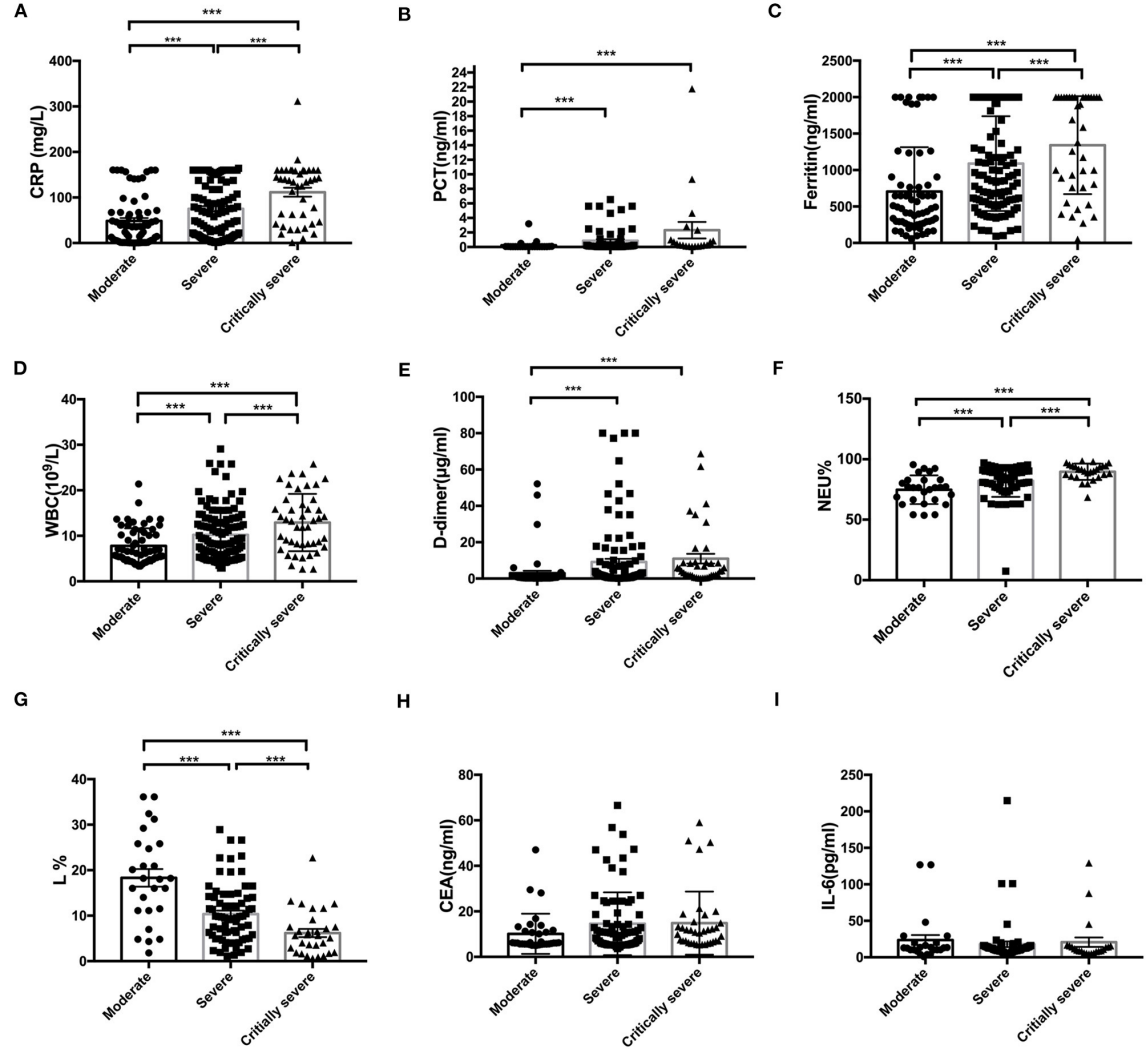
Correlation between the initial levels of CRP, PCT, Fer, WBC, D2, Neu%, L%, CEA, and IL-6 and clinical classification. **(A–F)** The levels of CRP, PCT, Fer, D2, WBC counts, and Neu percentage were significantly higher in the critically severe patients (*n* = 76) and severe patients (*n* = 155) than in the moderate patients (*n* = 83) (*P* < 0.05). **(G)** The L percentage was significantly lower in severely and critically severely affected patients than in moderately affected patients (*P* < 0.001). **(H,I)** No significant differences in the levels of CEA and IL-6 between the critically severe or severe patients and moderate patients were observed from the time of admission. CRP, C-reactive protein; PCT, procalcitonin; Fer, ferritin; WBC, white blood cell; D2, D-dimer; Neu%, neutrophils percentage; L%, lymphocyte percentage; CEA, Carcinoembryonic antigen; IL-6, interleukin 6. ***P < 0.05.

The correlations between the CRP level, WBC count, L count, and clinical classification are shown in Figure 1. In the critically severely affected patients (*n* = 76), CRP levels were significantly higher compared with those in moderately affected patients (*n* = 83) (*P* < 0.001) and severely affected patients (*n* = 155) (*P* = 0.001). The levels of PCT in severely and critically severely affected patients were significantly higher compared with those in moderately affected patients (*P* = 0.037, *P* = 0.002, respectively). The levels of Fer and the WBC counts in critically severely affected patients were significantly higher compared with those in moderately affected patients (*P* < 0.001). The levels of D2 in severely and critically severely affected patients were higher than those in moderately affected patients (*P* = 0.017, *P* = 0.004, respectively). The L% values in severely and critically severely affected patients were lower compared with those in moderately affected patients (*P* < 0.001). The Neu% values in severely and critically severely affected patients were higher (*P* = 0.002, *P* < 0.001, respectively). CEA and IL-6 levels were not associated with the clinical classification of COVID-19: no significant differences were seen between the three types of patients. These results suggested that the levels of CRP, PCT, Fer, D2, WBC counts, Neu%, and L% correlated closely with disease classification.

### The Critical Values of CEA, IL-6, CRP, PCT, Fer, D-Dimer Levels, L%, Neu%, and WBC to Assess COVID-19 Classification

**Figure 2:**
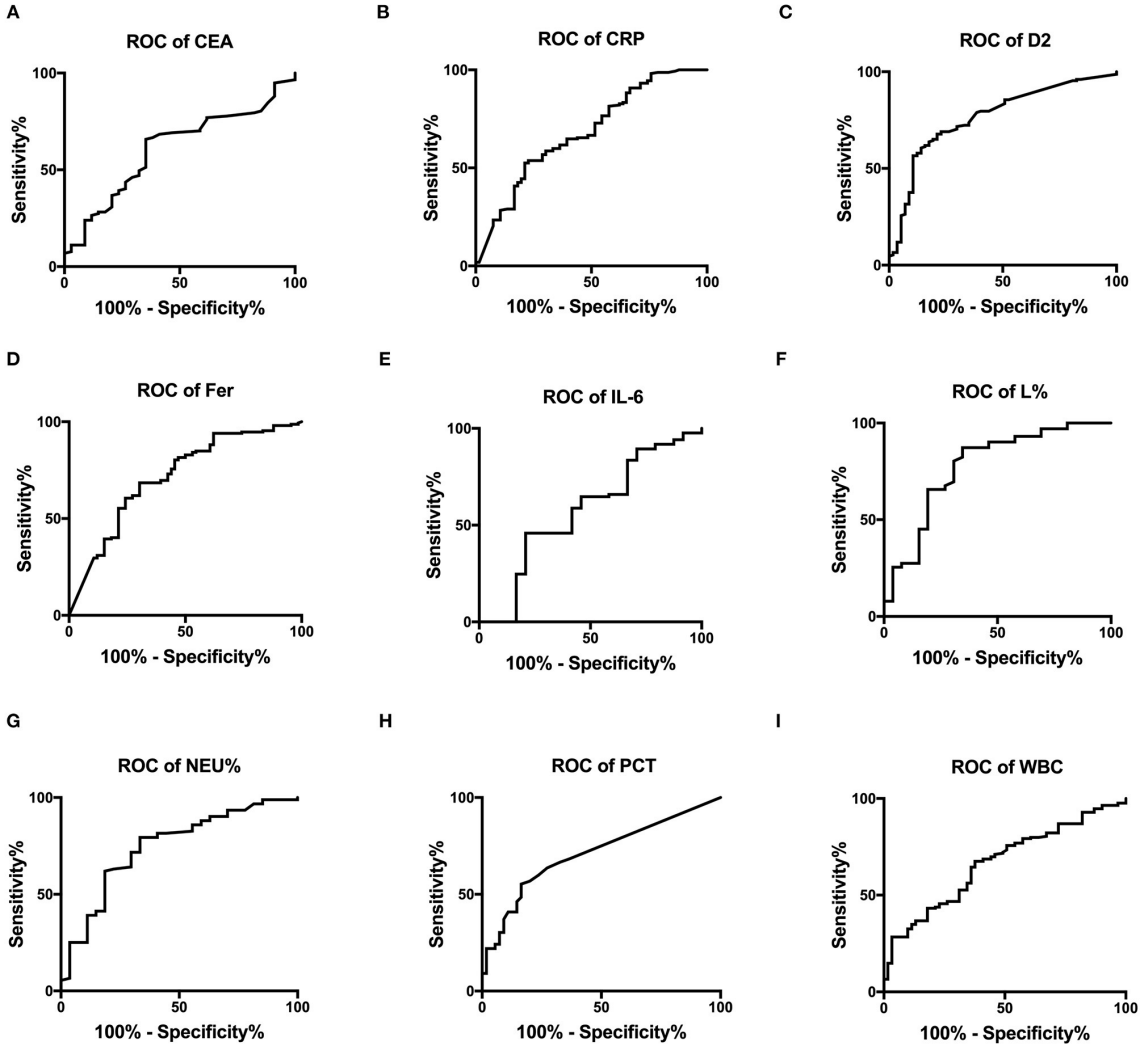
The ROC curves for CEA, CRP, D2, Fer, IL-6, L%, Neu%, PCT, and WBC were analyzed to assess disease classification. **(A–I)** ROC, receiver operating characteristic; AUC, area under the curve; CRP, C reactive protein; PCT, procalcitonin; Fer, ferritin; WBC, white blood cell; D2, D-dimer; Neu%, neutrophils percentage; L%, lymphocyte percentage; CEA, Carcinoembryonic antigen; IL-6, interleukin 6.

Figures 2A–I show the ROC curves for CEA, IL-6, CRP, PCT, Fer, D-dimer levels, L%, Neu%, and WBC, which were used to evaluate disease classification. For these markers, the area under the curve (AUC) values were determined as (from high to low): L% (0.776 ± 0.057) > D2 (0.766 ± 0.037) > Neu% (0.746 ± 0.055) > Fer (0.716 ± 0.039) > PCT (0.709 ± 0.039) > CRP (0.680 ± 0.04) > WBC (0.665 ± 0.038) > CEA (0.607 ± 0.053) > IL-6 (0.573 ± 0.072). The critical values for these markers to predict severe disease type were L% < 4.2%, Neu% > 92.6%, PCT > 0.795 ng/ml, D2 > 8.18 μg/ml, WBC > 13.76 × 10^9^/L, Fer > 907.4 ng/ml, CEA > 33.45 ng/ml, CRP > 102.8 mg/L, IL-6 > 10.21 pg/ml. According to the ROC curve analysis, we regarded the moderate type as negative and regarded severe and critically severe as positive.

### Correlations Between CEA, IL-6, CRP, PCT, Fer, D-Dimer, L%, Neu%, and WBC Levels and COVID-19 Prognosis

**Figure 3:**
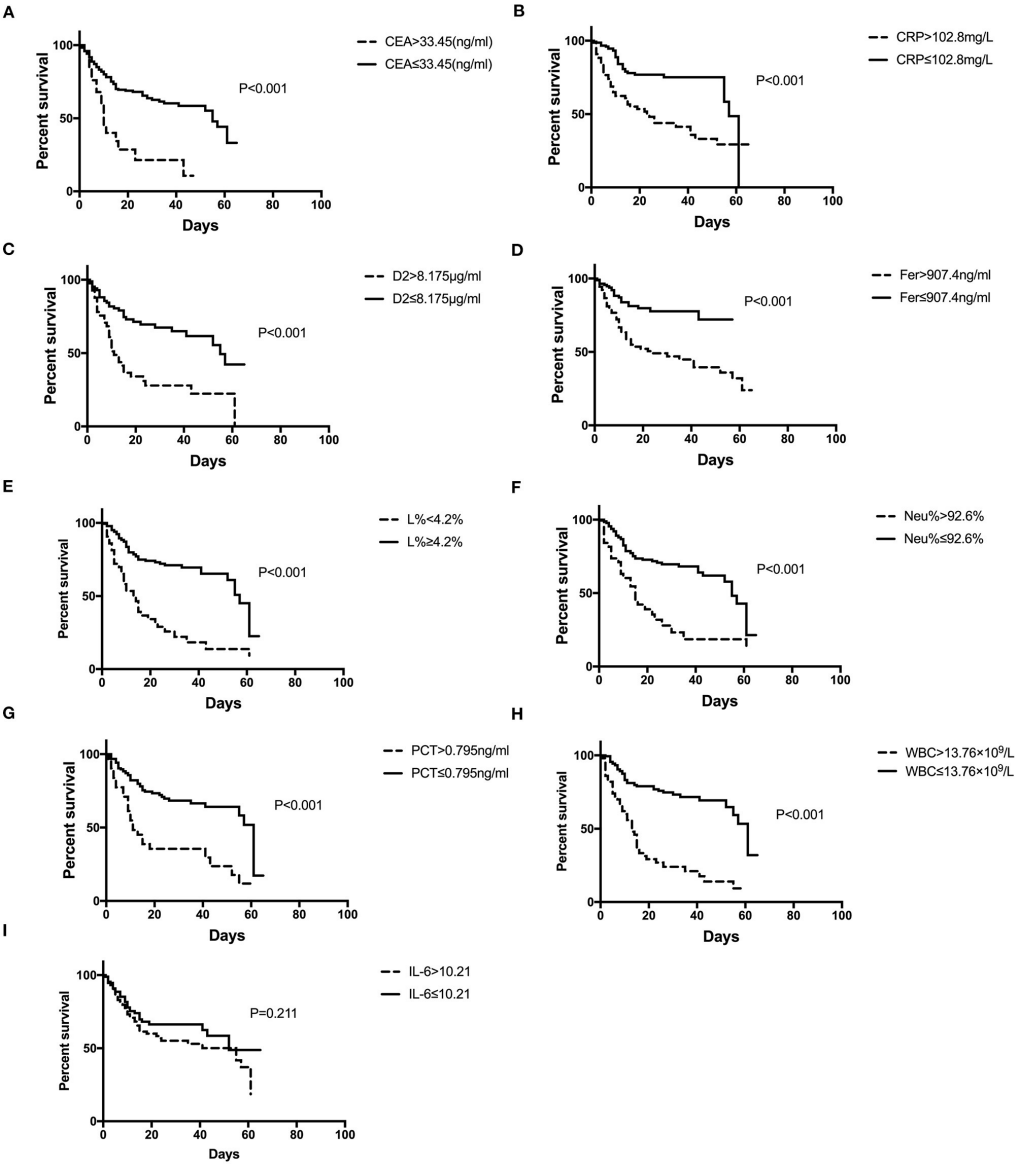
Survival curves constructed for the different initial levels of CEA, CRP, D2, Fer, IL-6, L%, Neu%, PCT, and WBC among the patients. **(A–D, F–H)** Patients with COVID-19 with initial CEA levels >33.45 ng/mL, CRP over 102.8 mg/L, D2 over 8.18 μg/ml, Fer over 907.4 ng/ml, Neu% over 92.6%, PCT levels >0.795 ng/ml, and WBC counts over 13.76 × 10^9^/L had poorer outcomes than those with lower levels, while patients with L% <4.2% had poorer outcomes **(E)**. **(I)** Patients with IL-6 levels higher or lower than 10.21 pg/ml showed no difference in outcome. COVID-19, Coronavirus disease 2019; CRP, C-reactive protein; PCT, procalcitonin; Fer, ferritin; WBC, white blood cell; D2, D-dimer; Neu%, neutrophils percentage; L%, lymphocyte percentage; CEA, Carcinoembryonic antigen; IL-6, interleukin 6.

**Figure 4:**
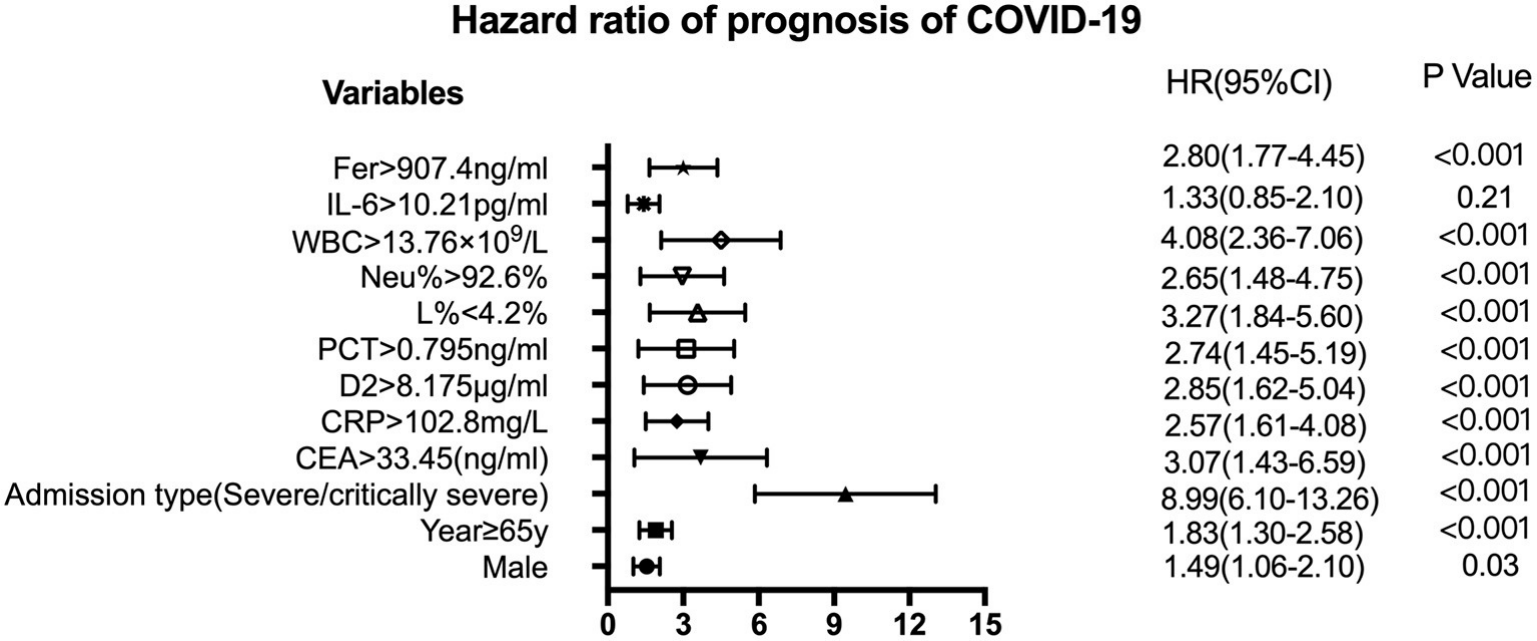
Forest plots of CEA, CRP, D2, Fer, IL-6, L%, Neu%, PCT, and WBC levels with other factors to assess the HR of the prognosis of COVID-19. The variables shown on the left of the axis, while the *P*-values are shown to the right of the HR. CI, confidence interval; HR hazard ratio; COVID-19, Coronavirus disease 2019; CRP, C-reactive protein; PCT, procalcitonin; Fer, ferritin; WBC, white blood cell; D2, D-dimer; Neu%, neutrophils percentage; L%, lymphocyte percentage; CEA, Carcinoembryonic antigen; IL-6, interleukin 6.

Figure 3 shows the survival curves for patients with COVID-19 with varying CEA, IL-6, CRP, PCT, Fer, D-dimer, L%, Neu% levels, and WBC counts at admission. Patients with initial CEA levels in excess of 33.45 ng/mL, WBC counts in excess of 13.76 × 10^9^/L, Neu% in excess of 92.6%, PCT levels in excess of 0.795 ng/ml, CRP levels in excess of 102.8 mg/L, Fer levels in excess of 907.4 ng/mL, and D2 levels in excess of 8.175 μg/ml displayed poorer prognosis compared with that of patients with lower amounts of these markers (Figures 3A–G). While patients with an initial L% < 4.2% had worse outcomes (Figure 3H). However, there were no differences in the prognosis of patients with IL-6 levels over 10.21 pg/mL (Figure 3I). Table 2 shows the effects of these markers on OS, as assessed using univariate and multivariate Cox regression analysis. The Forest plots of these markers and other factors (age, sex, and admission type) are shown in Figure 4. The hazard ratio and 95% confidence interval (CI) of the variables (Fer > 907.4 ng/ml, IL-6 > 10.21 pg/ml, WBC > 13.76 × 10^9^/L, Neu% > 92.6%, L% < 4.2%, PCT > 0.795 ng/ml, D2 > 8.18 μg/ml, CRP > 102.8 mg/L, and CEA > 33.45 ng/ml, along with the admission type, age, and sex) were 2.80 (1.77–4.45), 1.33 (0.85–2.1), 4.08 (2.36–7.06), 2.65 (1.48–4.75), 3.27 (1.84–5.6), 2.74 (1.45–5.19), 2.85 (1.62–5.04), 2.57 (1.61–4.08), 3.07 (1.43–6.59), 8.99 (6.10–13.26), 1.83 (1.3–2.58), and 1.49 (1.06-2.1), respectively. Most variables showed significant differences (*P* < 0.05), except for IL-6 > 10.21 pg/ml (*P* = 0.21). Thus, for the OS of patients with COVID-19, the independent prognostic risk factors comprised CEA, WBC, CRP, PCT, Fer, D-dimer, Neu%, and L%.

**Table 2:** Univariate and multivariate Cox proportional hazards regression analysis for overall survival (OS).

**Variables**	**Univariate**			**Multivariate**		
	**HR**	**95 CI**	***P*-values**	**HR**	**95 CI**	***P*-values**
Gender	1.49	1.06–2.10	0.03			
F	Ref					
Age	1.83	1.30–2.58	<0.001	2.63	1.14–6.08	0.006
<65	Ref					
Admission type	8.99	6.10–13.26	<0.001	2.29	1.27–4.14	0.024
Moderate	Ref					
Fer	2.80	1.77–4.45	<0.001	2.70	1.61–4.42	0.001
≤ 907.4 ng/ml	Ref					
IL-6	1.33	0.85–2.10	0.21			
≤ 10.21 pg/ml	Ref					
WBC	4.08	2.36–7.06	<0.001	2.19	1.08–4.44	0.003
≤ 13.76 × 10^9^/L	Ref					
Neu%	2.65	1.48–4.75	<0.001	2.53	1.60–4.03	0.001
≤ 92.6%	Ref					
L%	3.27	1.84–5.60	<0.001			
≥4.2%	Ref					
PCT	2.74	1.45–5.19	<0.001			
≤ 0.795 ng/ml	Ref					
D2	2.85	1.62–5.04	<0.001	2.22	1.13–4.35	0.021
≤ 8.175 μg/ml	Ref					
CRP	2.57	1.61–4.08	<0.001			
≤ 102.8 mg/L	Ref					
CEA ≤ 33.45 ng/ml	3.07 Ref	1.43–6.59	<0.001	2.00	1.19–3.35	0.009

### The Prognostic Nomogram for OS

**Figure 5:**
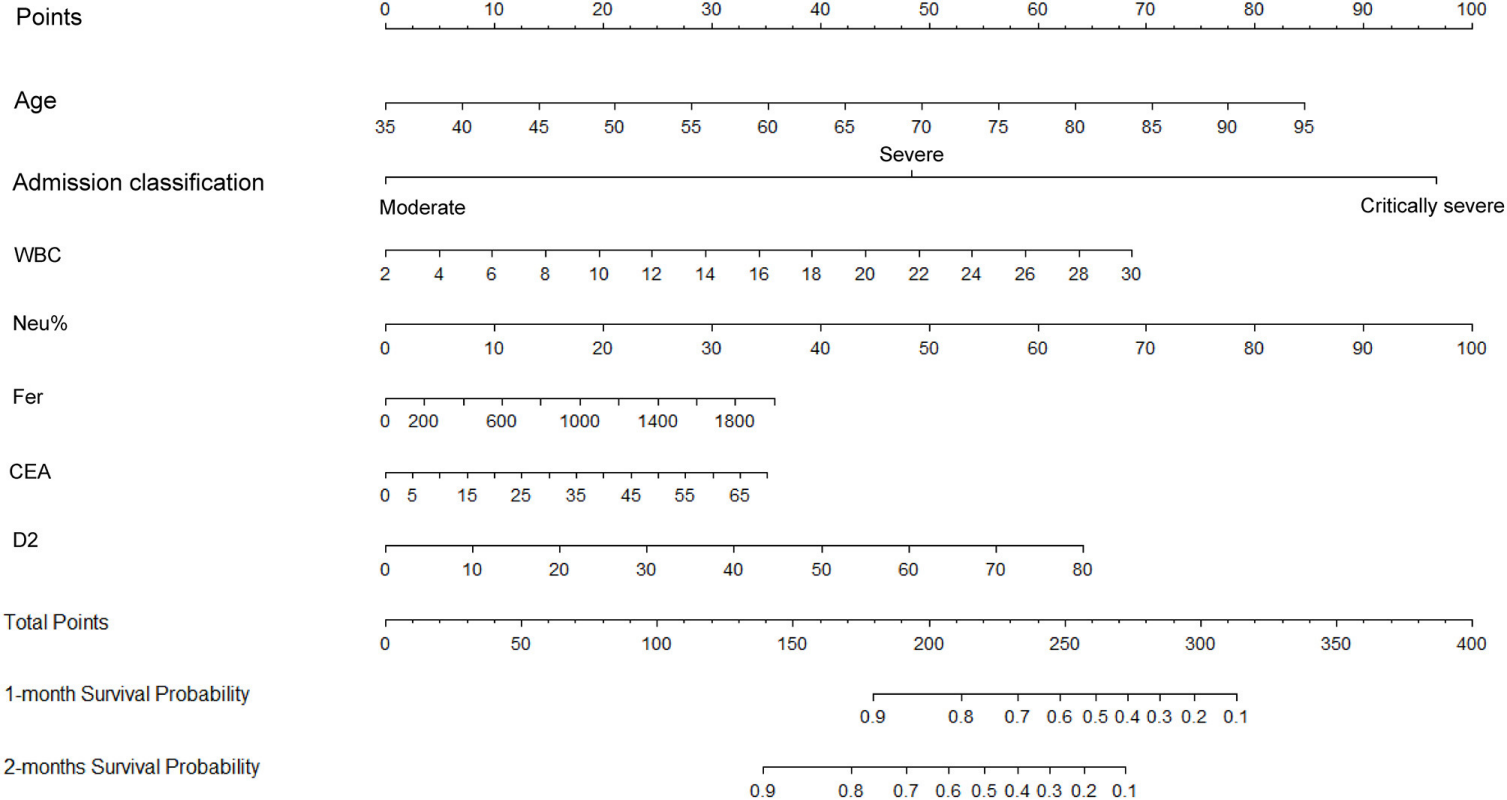
Construction of a nomogram to predict the overall survival of patients with COVID-19 comprising CEA levels and other significant indicators. The points total are located on the Total Point axis, and a vertical line is traced downward to the survival axes to predict the likelihood of an OS of 1 or 2 months. COVID-19, Coronavirus disease 2019; CEA, Carcinoembryonic antigen.

The independent indicators from the multivariate analysis were used to construct the prognostic nomogram for OS of patients with COVID-19 (Figure 5). Compared with that of the other variables, for the outcome in patients with COVID-19, the prognostic value of Neu% was more significant (*P* < 0.001). In order of importance, the remaining factors were Fer (*P* = 0.000), CEA (*P* = 0.000), D2 (*P* = 0.000), WBC (*P* = 0.000), CRP (*P* = 0.000), and PCT (*P* = 0.000), while the nomogram model was not affected significantly by IL-6 (*P* = 0.21; Table 2). In the nomogram, each predictor was given a score (top scale), the sum of which indicated the probability of OS for 1 or 2 months (bottom scale). For OS, the nomogram had a C-index of 0.84 (95% CI, 0.79–0.88), demonstrating that the model had a good discriminative ability (admission classification + WBC + Neu% + Fer + CEA + D2, Figure 5).

### The OS Nomogram Model Calibration Curves

**Figure 6:**
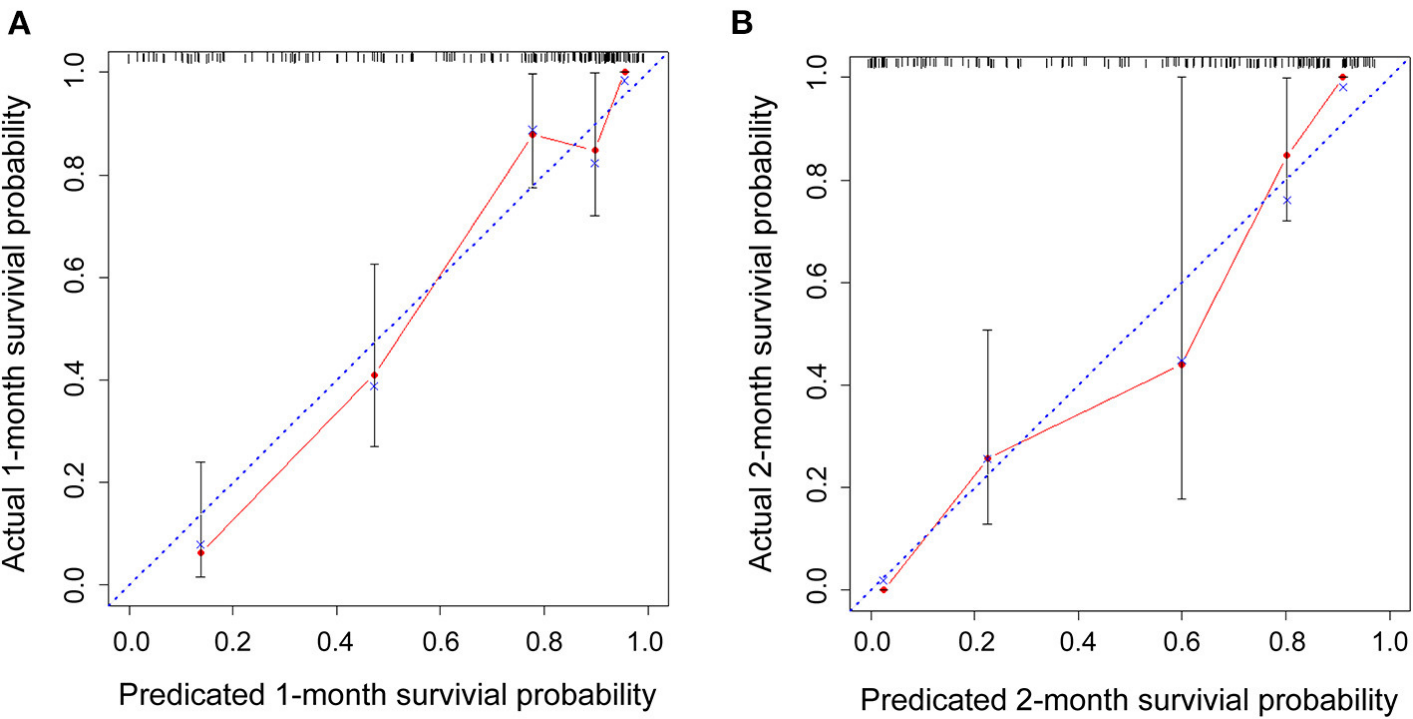
Internal cross-validation calibration curves at 1 and 2 months. **(A,B)** Internal cross-validation calibration plots at 1 and 2 months.

Figure 6 displays the calibration curves for internal validation at 1 and 2 months. For the internal cross-validation, the calibration plots for 1 and 2 months closely approximated to the observed estimates (Figures 6A,B). For OS for 1 and 2 months, the AUC values were 0.87 (95% CI, 0.81–0.94) and 0.83 (95% CI, 0.76–0.89), respectively.

## Discussion

Since the COVID-19 outbreak, SARS-CoV-2 infection has resulted in more than 40 million infections and over 1 million deaths worldwide. The infected patients may develop acute respiratory distress syndrome and die rapidly from a series of complications, including acute inflammation, coagulation dysfunction, septic shock, and multiple organ failure, which is especially the case for elderly patients with underlying diseases (5, 12). The severe disease-related complications and diverse clinical characteristics mean that early diagnosis and treatment can improve prognosis and reduce mortality in patients with COVID-19 (1, 13).

COVID-19 severity is associated with the levels of CEA, IL-6, CRP, PCT, Fer, D-dimer, L%, Neu%, and WBC. Here, we found that the critical values for those indicators were: L% < 4.2%, Neu% > 92.6%, PCT > 0.795 ng/ml, D2 > 8.18 μ g/ml, WBC > 13.76 × 10^9^/L, Fer > 907.4 ng/ml, CEA > 33.45 ng/ml, CRP > 102.8 mg/L, IL-6 > 10.21 pg/ml, respectively. The AUC values for these markers (from ROC curve analysis) from high to low were L% (0.776 ± 0.057) > D2 (0.766 ± 0.037) > Neu% (0.746 ± 0.055) > Fer (0.716 ± 0.039) > PCT (0.709 ± 0.039) > CRP (0.680 ± 0.04) > WBC (0.665 ± 0.038) > CEA (0.607 ± 0.053) > IL-6 (0.573 ± 0.072). Thus, clinicians should monitor changes in these indicators during patient treatment. Increased CEA, Fer, PCT, D2, CRP levels, Neu%, and WBC counts indicate severe pneumonia, while decreased levels indicate treatment effectiveness and disease improvement. However, an increased L% indicates disease improvement, while decreased ratios indicate disease progression. Furthermore, our data show that CEA levels decreased below 5 ng/mL in well-recovered patients. CRP, WBC count, L%, Neu%, PCT, IL-6, and Fer are inflammatory markers commonly used to evaluate the inflammatory state of patients. D-dimer is a marker of thromboembolism (13–15). Studies have demonstrated that an increased level of D2 indicates a high risk for venous thromboembolism in patients with COVID-19. The levels of CRP, Fer, PCT, and IL-6, an acute phase protein, increase in the body immediately in response to infection or tissue damage (16, 17). This results in the activation of the complement system and strengthening of the phagocytic cell-mediated defense against invading microorganisms. WBCs and Ls are the major immune cells that rapidly initiate immune responses when the body is infected with a virus (18).

The serum CEA level has been identified as a prognostic marker for HIV-related pneumocystis carinii pneumonia (PCP) (19), in which patients with PCP and acute respiratory distress have increased CEA levels. Moreover, fatal outcomes were only associated with high concentrations of CEA (> 20 ng/mL) in patients with a PaO_2_ value lower than 50 mmHg (19, 20). The results of the present study also showed that patient outcome in COVID-19 is associated with preliminary CEA levels.

In our study, we constructed an OS nomogram for the clinical diagnosis and treatment of COVID-19 with the models (Admission classification + WBC + Neu% + Fer + CEA + D2), and the nomogram of OS had a C-index of 0.84 (95% CI, 0.79–0.88). The model could be used to assess the clinical risk factors to predict the OS of patients with COVID-19. Furthermore, the calibration plots for the internally cross-validated cohort closely approximated to the observed estimates. From the prognostic risk score, we could identify the populations of patients at high risk of shorter OS and provide effective treatment for a better outcome. According to the hazard ratio for the prognosis of risk variables for COVID-19, the admission classification (severe or critically severe), age over 65 years old, levels of Fer over 907.4 ng/ml, PCT over 0.795 ng/ml, D2 over 8.175 μg/ml, CRP over 102.8 mg/L, CEA over 33.45 ng/ml (excluding tumors), a WBC count over 13.76 × 10^9^/L, Neu% over 92.6%, and L% below 4.2% were higher risk factors for poor patient OS. However, our data showed no significant difference in the HR between different levels of IL-6. In conclusion, our study provided a nomogram model comprising clinical biomarkers, such as Fer, PCT, CRP, D-dimer, and CEA. These data will provide useful information for the diagnosis, management, and therapy of COVID-19.

## Data Availability Statement

The original contributions presented in the study are included in the article/supplementary materials, further inquiries can be directed to the corresponding author/s.

## Author Contributions

JY, LN, and XZ had access to all the clinical data generated by the study, took responsibility for data integrity, accuracy of the data analysis, concept, and design. DW and JC: acquisition, analysis, or interpretation of data. JY and LN: manuscript preparation. ZY and LZ: statistical analysis. DL: supervision. All authors contributed to the article and approved the submitted version.

## Conflict of Interest

The authors declare that the research was conducted in the absence of any commercial or financial relationships that could be construed as a potential conflict of interest.
